# Bioremediation of malachite green dye using sodium alginate, *Sargassum latifolium* extract, and their silver nanoparticles

**DOI:** 10.1186/s13065-023-01022-0

**Published:** 2023-08-31

**Authors:** Mostafa M. El-Sheekh, Mohamed A. Deyab, Nagwa I. Hassan, Seham E. Abu Ahmed

**Affiliations:** 1 Botany Department, Faculty of Science, Tanta University, Tanta, 31527 Egypt; 2 Botany and Microbiology Department, Faculty of Science, Damietta University, Damietta El-Gededa, 34517 Egypt

**Keywords:** Sodium alginate, Silver nanoparticles, Malachite green dye, *Sargassum latifolium*, Adsorption isotherm, UV and FT-IR

## Abstract

**Introduction:**

The textile, paper, rubber, plastic, leather, cosmetics, pharmaceutical, and food sectors extensively use malachite green (MG). In spite of this, it has mutagenic, carcinogenic, teratogenic, and, in some circumstances causes chronic respiratory disease.

**Objectives:**

In this work, we used sodium alginate, *Sargassum latifolium* aqueous extract, and their silver nanoparticles to test their potential as inexpensive adsorbent agents to remove malachite green dye from aqueous solutions.

**Methods:**

The removal rate of MG was determined using a series of bioadsorption experiments. Besides, the effect of different factors on bioadsorption, such as pH, adsorbent dose, contact time (min), and different concentrations of MG dye was investigated.

**Results:**

The removal efficiency of MG dye by alginate nanoparticles, alginate, *Sargassum latifolium* aqueous extract, and *S. latifolium* aqueous extract nanoparticles was 91, 82, 84, and 68 respectively. The optimal conditions for bioadsorption of malachite green dye were pH 7, a contact time of 180 min, and an adsorbent dose of 0.02 g. The adsorption isotherm was fitted to Langmuir and Freundlich isotherm. Also, UV and FT–IR before and after the bioadsorption of MG were performed to confirm the bioadsorption process.

**Conclusion:**

Our results indicated that alginate nanoparticles were the most effective bioadsorbent agent.

## Introduction

The typical molecular structure of dyes is complex and aromatic, which increases their stability and makes biodegradation more difficult. The widespread use of malachite green (MG) in the textile industry is crucial for both human health and aquatic environments worldwide because of the huge discharges of untreated wastewater containing this dye. High concentrations of harmful and resistant compounds, including dyes, are present in textile industry effluents, which negatively impact the environment and, ultimately, people [1, 2]. According to [3], fish that live in MG-contaminated water for just one day can maintain its toxic form by keeping MG in the body even after living in fresh water for a week. As a result of the very long-lasting residual presence in the treated fish, this highly toxic dye can easily pass to humans via the food chain [4]. The low concentrations of synthetic organic dyes in water sources led to the initial classification of these substances as microcontaminants [5]. However, monitoring programmers show that dye contamination is quickly becoming a matter of environmental concern. About 10–15% of the dye from industry is directly released into the environment during the dyeing process, which could disrupt the ecosystem [6, 7]. They build up throughout the aquatic wildlife’s food chain and interfere with the physiological processes of aquatic flora by blocking their pathways for photosynthetic activity. As a result, aquatic ecosystems lack adequate oxygen circulation and light absorption [2]. MG is widely used in the fish farming business as a fungicide, anti-protozoan ectoparasites, and disinfection, as well as for colouring cotton, jute, silk, wool, and leather [6, 8, 9]. On the other hand, malachite green has been linked to an increased risk of cancer and stimulates liver tumors in mammalian cells [10, 11].

As a result, removing these colors from industrial discharges before they are dumped into the environment is critical. To manage dye-contaminated water, many researchers developed a variety of treatment technologies (chemical, physical, and biological). Due to its simplicity of design and great efficiency, the bioadsorption process is a preferred method among all those that are now accessible [12, 13].

This paper focused on the effect of different factors (contact time, pH, adsorbent dose, and various concentrations of malachite green dye) on the bioremoval of malachite green dye by alginate, *Sargassum latifolium* aqueous extract and their silver nanoparticles from aqueous solutions. Langmuir and Freundlich's adsorption isotherm were also studied.

## Materials and methods

1. Collection and identification of the macroalga: The brown seaweed *Sargassum latifolium* (Turner) C.Agardh, was collected from Ras Sudr shores, Red Sea Coast, Egypt. *S. latifolium* was identified using preserved herbarium sheets in the Department of Botany, Faculty of Science, Damietta University, Egypt. The identification was also confirmed by using Algae Base (https://www.algaebase.org/).
2. The sodium alginate from *S. latifolium* extracted in water-soluble salts and the formation of its silver nanoparticles occurred by dissolving alginate in 1 mM AgNO_3_ and the method mentioned in detail in our previous research [14]. The formation of *S. latifolium* aqueous extract was prepared by combining 1 g of algal powder with 100 ml of distilled water and the details were also studied in our previous research [15]. The formation of its silver nanoparticles was the same as the alginate nanoparticles preparation.

## Preparation of dye

Malachite green is a cationic dye, soluble in water, and appears as a green crystalline powder. A stock solution of 1000 mg L^−1^ of MG dye was prepared. To obtain the needed solutions, this solution was diluted (20, 50, 100, 150, and 200 mg/l). The maximum absorption peak for malachite green dye was determined using UV—visible spectral analysis between 200 and 800 nm.

## Batch adsorption experiments

### How the starting pH affects the biodegradation of the malachite green dye

The current experiment was carried out in 250 ml flasks with a specific concentration of 10 mg of biosorbent in a 100 ml MG dye solution. The pH of the combination was adjusted using 0.1 N·HCL and 0.1 N·NaOH solutions to 4.0, 6.0, 7.0, and 8.0. For four hours, the flasks were shaken at 200 rpm at room temperature. After centrifuging the samples for 20 min at 4000 rpm, the remaining MG molecules were identified using a spectrophotometer. The biosorption capacity can be estimated by the following equation:

$${\text{Biosorption capacity Q }}\left( {{\text{mg}}/{\text{g}}} \right) \, = {\text{V }}\left( {{\text{C}}_{0} - {\text{ C}}_{{\text{e}}} } \right)/{\text{W}}$$ [16]

C_0_ is the initial dye concentration, C_e_ is the final concentration of dye in the solutions (mg), V is the volume of the solution (L), and W is the biosorbent mass (g).

The removal rate was evaluated as follows:

$${\text{Removal rate }}\left( \% \right) \, = \, \left( {{\text{C}}_{0} - {\text{ C}}_{{\text{e}}} } \right) \, /{\text{ C}}_{0} \times { 1}00$$

where C_0_ was the initial dye concentration (mg), C_e_ was the residual dye concentration in solution (mg).

### Effectiveness of contact time

The experiment was carried out in 250 ml flasks with a specific concentration of 10 mg of biosorbent in a 100 ml MG dye solution. The solution’s pH was kept constant at 7.0. (obtained from the previous). For four hours, the flasks were shaken at 200 rpm at room temperature. The centrifugation of samples (4000 rpm, 20 min) every 30 min, 60 min, 90 min, 120 min, 150 min, 180 min, 210 min, and 240 min, then the residual malachite green dye in the solutions was estimated using a spectrophotometer.

### Effectiveness of biosorbent dose

The experiment was carried out in 250 ml flasks with 100 ml of MG solution and initial concentrations of 5, 10, 15, 20, and 30 mg of biosorbent. The medium’s pH was adjusted to 7.0. (obtained from the previous experiment). For 2.30 h, the flasks were shaken at 200 rpm at room temperature (obtained from the previous experiment). The residual malachite green molecules in solutions were calculated using a spectrophotometer after samples were centrifuged (4000 rpm, 20 min). Using [16] estimates, the biosorption capacity and elimination rate.

### Effectiveness of initial malachite green dye concentrations

In 250 ml flasks with a certain quantity of biosorbent (20 mg) obtained from the prior experiment, the experiment was carried out. The medium’s pH was adjusted to 7.0. (obtained from the previous experiment). For three hours, the flasks were shaken at 200 rpm at room temperature (obtained from the previous experiment). The amount of remaining malachite green molecules in the solutions was calculated after centrifuging the samples for 20 min at 4000 rpm. Using [16] estimates, the biosorption capacity and elimination rate. Data acquired with triplicate trails was used to compute the mean values and standard errors. The mean values and the standard errors were calculated from the data obtained with triplicate trails.

## UV scan analysis for metal ion concentrations

UV scan for 100 mg/L MG with and without alginate nanoparticles was done by ANTI UNICAM-UV VISIBLE VISION SOFTWARE V3.20 after an incubation period (150 min) to confirm the bioadsorption of dye.

## Fourier transform infrared spectrometry (FT-IR) for metal ion concentrations

FT–IR analysis was done using the Mattson 5000 FT-IR spectrometer to illustrate the variation in the functional group of alginate nanoparticles after the adsorption of dye.

## Adsorption isotherms

One crucial physicochemical factor in understanding the behaviour of bioadsorption in a solid-liquid system is the equilibrium of sorption. Two well-known models, the Freundlich and Langmuir isotherms, are used in the current work [17].

## Results and discussion

### UV scan analysis of Malachite green dye

**Fig. 1:**
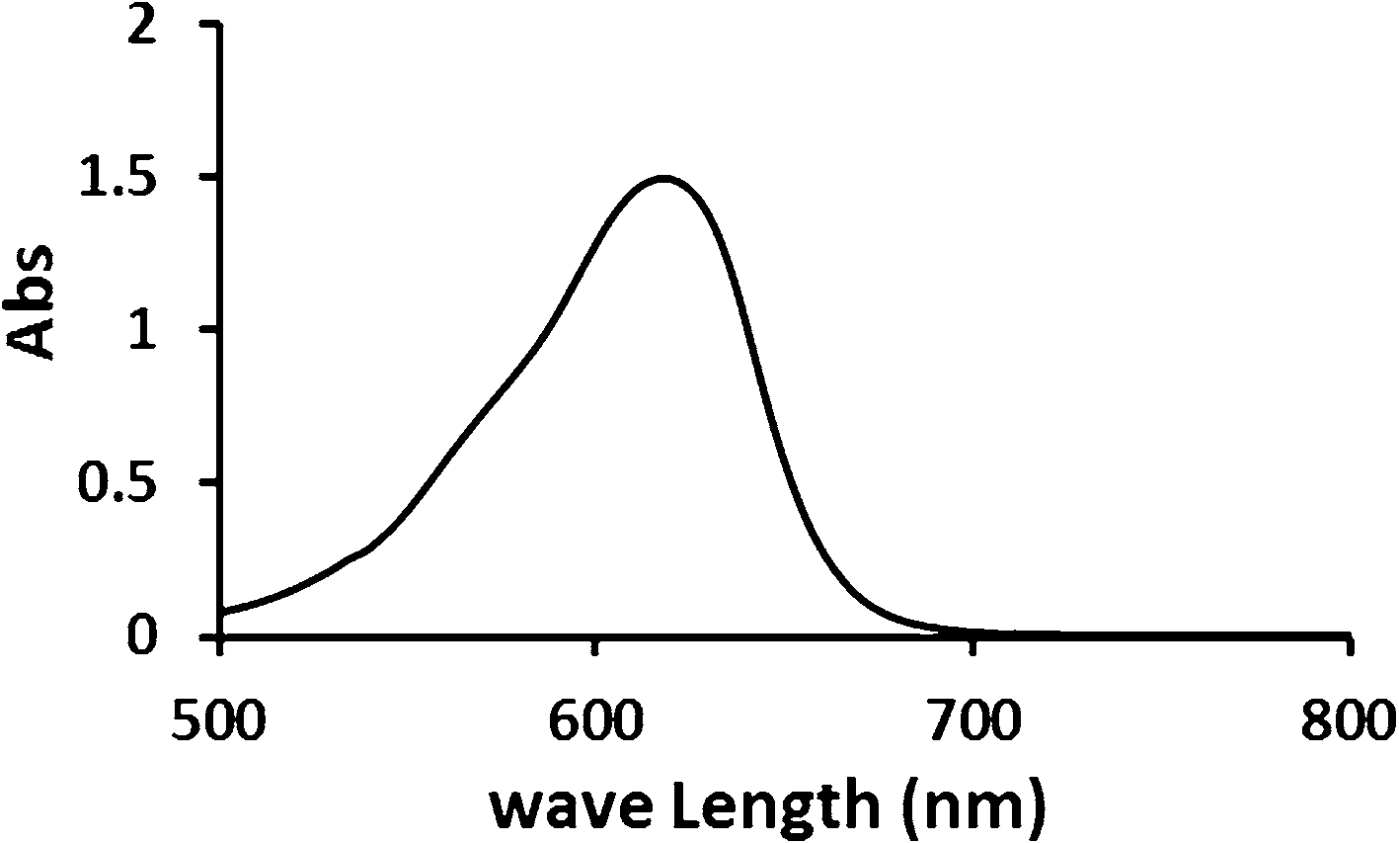
UV–vis absorbance spectrum of malachite green dye maximum absorption peak

According to Fig. 1 the maximum absorption peak (λ _max_) of malachite green dye is 615 nm. The findings suggested that biosorption alone may roughly account for eliminating malachite green [18].

## Bioadsorption experiments of malachite green dye

### Effect of initial pH on biodegradation of malachite green dye (MG)

**Fig. 2:**
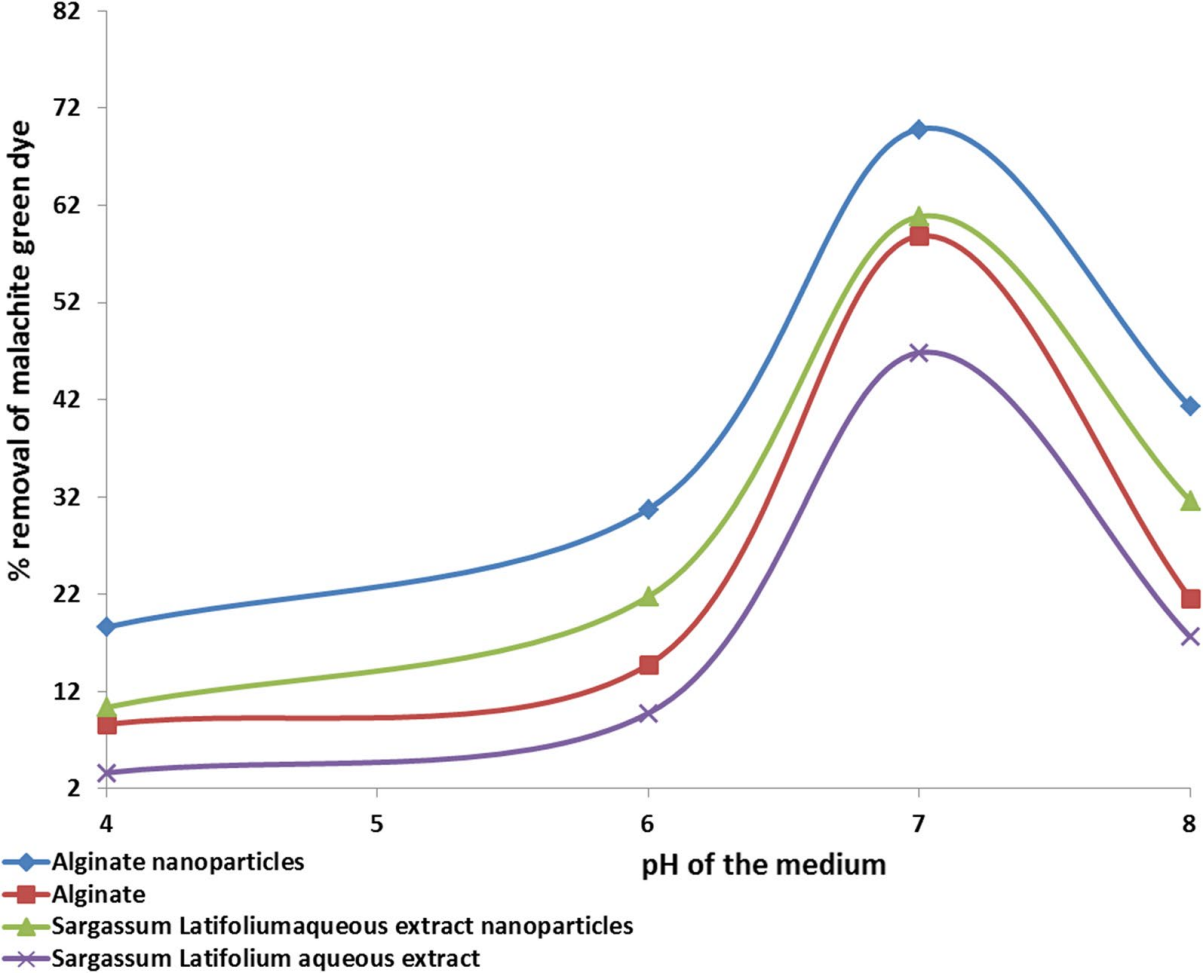
Effect of pH on bioadsorption of malachite green dye by alginate, alginate silver nanoparticles, *Sargassum latifolium* aqueous extract nanoparticles and aqueous extract

Ref. [19] reported that pH is an important factor in dye adsorption as it affects the speciation of dye, the ability of adsorbent, and surface charge, all of which influence dye-pollutant interactions. Furthermore, hydrogen ions compete with adsorbate ions for active sites on the adsorbent surface so that pH can alter dye molecule structure stability and color intensity. In the present study (Fig. 2), pH 4–8 was used to observe better adsorption with an initial concentration of dye 100 mg/L with a 10 mg adsorbent dosage. These findings were agreed with [20], who reported the best adsorption capacities of malachite green molecules were at pH 6 for *Zea mays*/Fe-Cu nanoparticles. Table 1 showed the most favorable adsorption of MG that seen at pH 7 with the percentage of removal by alginate silver nanoparticles (69.8 %) > S. *latifolium* aqueous extract nanoparticles (60.8%) > alginate (58.8%) > *S. latifolium* aqueous extract (46.8%) and also biosorption capacity (Q) of alginate silver nanoparticles (698.4 mg/g) > *S. latifolium* aqueous extract nanoparticles (608.4 mg/g) > alginate (588.4 mg/g) > aqueous extract (468.4 mg/g). Moreover, [22] found that the most favorable adsorption of malachite green by wood apple shell was obtained at pH from7 to 9 with a removal percent of 98.87. The reaction mixture was agitated for 240 min. Also, when the pH was raised from 4 to 8, the adsorption capabilities of nanoparticles increased. On the other hand, [22] found the maximum removal of malachite green dye using immobilized *Saccharomyces cerevisiae* at pH 5. Moreover, [23] noted the maximal adsorption of MG dye with sodium alginate hydrogel composite was found at pH 7 (89.71%) due to electrostatic interactions between positively charged dye molecules and negatively charged adsorbent. Also, our results are in accordance with [24], who stated that the removal rate of malachite green increased to pH 7 with a 73 percent removal rate.

**Table 1:** Effect of pH on bioadsorption of Malachite green dye by alginate, alginate silver nanoparticles, *Sargassum latifolium* aqueous extract nanoparticles and aqueous extract

pH	C_0_ (mg)	Treatments											
		Alginate nanoparticles (0.01 g)			Alginate (0.01 g)			*Sargassum latifolium* aqueous extract nanoparticles (0.01 g)			*Sargassum latifolium* aqueous extract (0.01 g)		
		C_e_ (mg)	Q (mg/g)	Removal rate	C_e_ (mg)	Q (mg/g)	Removal rate	C_e_ (mg)	Q (mg/g)	Removal rate	C_e_ (mg)	Q (mg/g)	Removal rate
4	100	81 ± 0.16	186 ± 1.5	18.6 ± 0.57	91 ± 0.95	86.5 ± 1.5	8.65 ± 0.15	89.6 ± 10	103 ± 1	10 ± 0.89	96 ± 10.5	36.5 ± 1.5	3.65 ± 0.15
6	100	69 ± 0.99	307 ± 0.95	30 ± 0.09	85 ± 4	147 ± 0.95	14 ± 0.09	78 ± 12	217 ± 40	21 ± 0.05	90 ± 9	97 ± 5	9.7 ± 0.9
*7*	*100*	*30* ± *0.12*	*698* ± *10*	*69.8* ± *0.5*	*41* ± *0.12*	*588* ± *11*	*58.8* ± *0.12*	*39* ± *2*	*608* ± *1.3*	*60.8* ± *9*	*53* ± *8*	*468* ± *1*	*46.8* ± *5*
8	100	58.7 ± 0.49	413 ± 4	41 ± 0.4	78 ± 10	216 ± 2	21.6 ± 0.23	68 ± 0.24	316 ± 2	31 ± 0.2	82 ± 10	176 ± 2	17 ± 2

C_o_: initial malachite green concentrations

C_e_: malachite green concentrations (ppm) after the incubation period (240 min)

Q: biosorption capacity

On the other hand, at low pH (1–3), there is a large concentration of hydrogen ions in the adsorbent, which compete with the cationic MG molecules for active sites, lowering dye adsorption effectiveness. Furthermore, the adsorbent is predominantly positively charged at low pH, resulting in significant repulsive interactions against positively charged dye species. On the other hand, this competition decreases when the pH rises from 4 to 7, and malachite green primarily occupies adsorbent sites. Discoloration of malachite green dye increases at higher pH because of the interaction between double bonds of MG molecules and hydroxyl groups in the solution [25–27]. The present study experiments were established at pH 7 to avoid the hydrolysis of malachite green dye. Also, [28] investigated the bioadsorption of malachite green by alginate nanoparticles at basic pH levels increased due to ionization and interaction of most of the functional groups of sodium alginate (COO^−^ and OH^−^) with the cationic dye molecules through a strong electrostatic interaction.

### Effect of contact time on the adsorption process

**Fig. 3:**
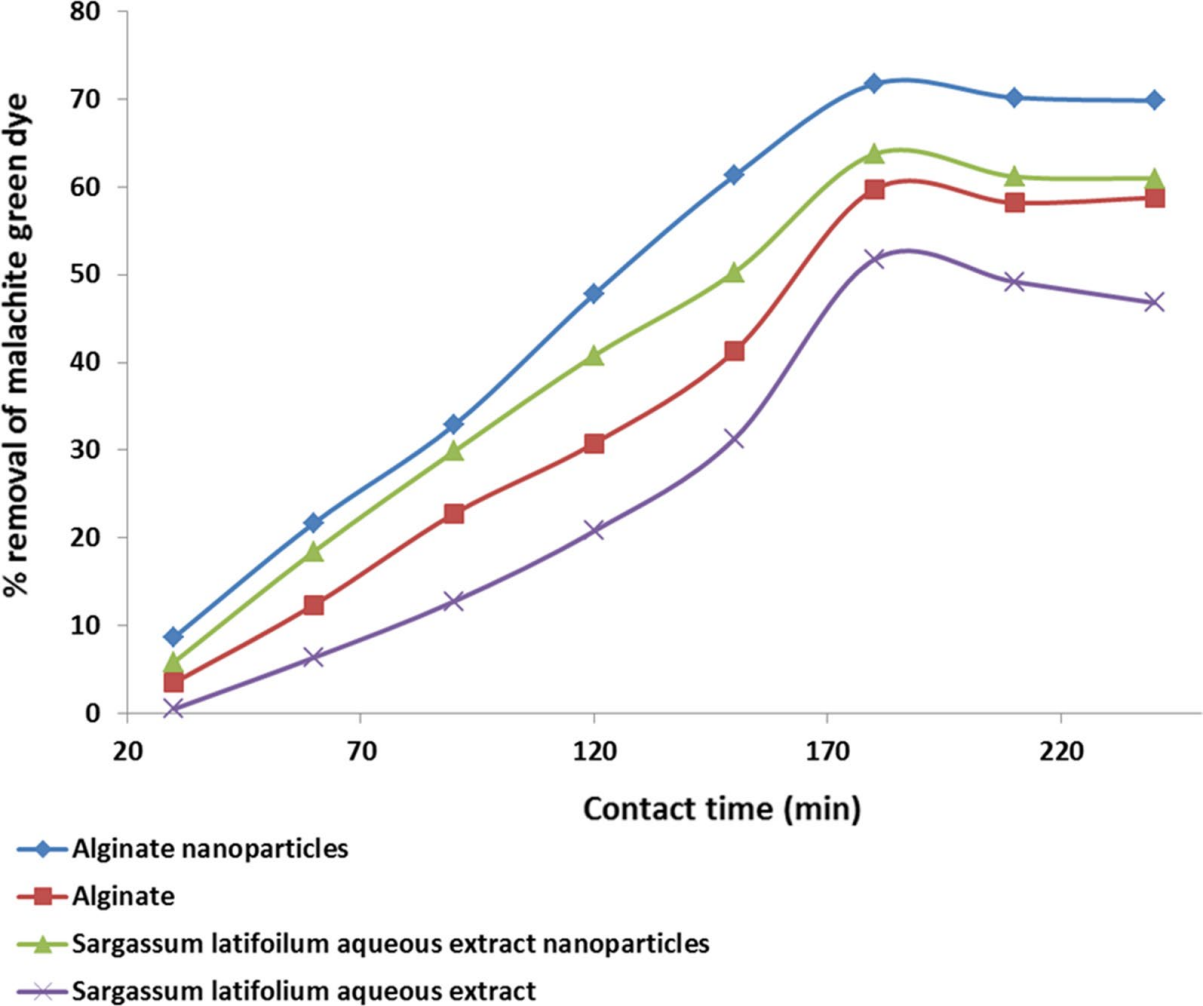
Effect of contact time on malachite green removal percentage by alginate, alginate silver nanoparticles, *Sargassum latifolium* aqueous extractnanoparticles and aqueous extract

In the present study, the percentage of dye removal as a function of time was investigated with an initial concentration of 100 mg/L of malachite green dye and an adsorbent dosage of 0.02 g. As shown in Fig. 3 and Table 2 the uptake of dye by adsorbents occurs at a faster rate at an equilibrium time of 180 min. The initial rate of adsorption was higher, but it gradually decreased; this could be attributed to the availability of a whole active site surface. Because the proportional increase in dye removal after 3 h was not significant, this was chosen as the best contact period. Our findings were in agreement with the earlier reports of [21, 29, 30]. A monolayer of adsorbate forms on the surface of the adsorbent in batch-type adsorption systems and the rate of removal of adsorbate molecules from the aqueous solution is primarily controlled by the rate of transport of the adsorbate molecules from the exterior/ outer sites to the interior site of the adsorbent particles [31].

**Table 2:** Effect of contact time on bioadsorption of malachite green dye by alginate, alginate silver nanoparticles, *Sargassum latifolium* aqueous extract nanoparticles and aqueous extract

Contact time (min)	C_o_ (mg)	Treatments											
		Alginate nanoparticles (0.01 g)			Alginate (0.01 g)			*Sargassum latifolium* aqueous extract nanoparticles (0.01 g)			*Sargassum latifolium* aqueous extract (0.01 g)		
		C_e_ (mg)	Q (mg/g)	Removal rate	C_e_ (mg)	Q (mg/g)	Removal rate	C_e_ (mg)	Q (mg/g)	Removal rate	C_e_ (mg)	Q (mg/g)	Removal rate
30	100	91 ± 0.17	86.6 ± 1	8.5 ± 1	96 ± 3	36 ± 1	3.5 ± 0.5	94 ± 5	56 ± 1	5.5 ± 0.7	98 ± 1.7	16 ± 2	0.5 ± 0.09
60	100	78 ± 1	217 ± 11	20 ± 3	88 ± 2	117 ± 10	12 ± 2	81 ± 5	183 ± 10	18 ± 2	93 ± 0.1	63 ± 1	6 ± 0.9
90	100	67 ± 2	328 ± 5	32 ± 5	77 ± 3	228 ± 5	22 ± 3	70 ± 9	298 ± 20	29 ± 3	87 ± 3	128 ± 6	13 ± 1.2
120	100	52 ± 1	477 ± 20	47 ± 9	69 ± 2	307 ± 10	30 ± 0.9	59 ± 5	407 ± 20	40 ± 6	79 ± 3	208 ± 15	21 ± 5
150	100	38 ± 2	612 ± 23	61 ± 5	58 ± 0.8	412 ± 15	41 ± 2	50 ± 7	492 ± 40	49 ± 5	69 ± 5	313 ± 20	31 ± 0.2
*180*	*100*	*28* ± *0.01*	*717* ± *18*	*71* ± *10*	*40* ± *0.5*	*597* ± *16*	*59* ± *0.7*	*36* ± *1*	*637* ± *50*	*63* ± *2*	*48* ± *6*	*518* ± *12*	*52* ± *5*
220	100	30.8 ± 1	691 ± 25	69 ± 2	41 ± 0.6	581 ± 20	58 ± 2	38 ± 1	611 ± 26	61 ± 5	51 ± 4	492 ± 26	49 ± 2
250	100	30.1 ± 0.12	698 ± 10	69 ± 5	41 ± 0.12	588 ± 12	58 ± 5	39 ± 0.12	608 ± 11	60 ± 9	53 ± 0.1	468 ± 12	47 ± 3

C_o_: intial malachite green dye concentrations pH: 7

C_e_: MG concentrations (mg) after incubation period (250 min) Q: biosorption capacity

### Effect of bioadsorbent dose on the bioadsorption process

**Fig. 4:**
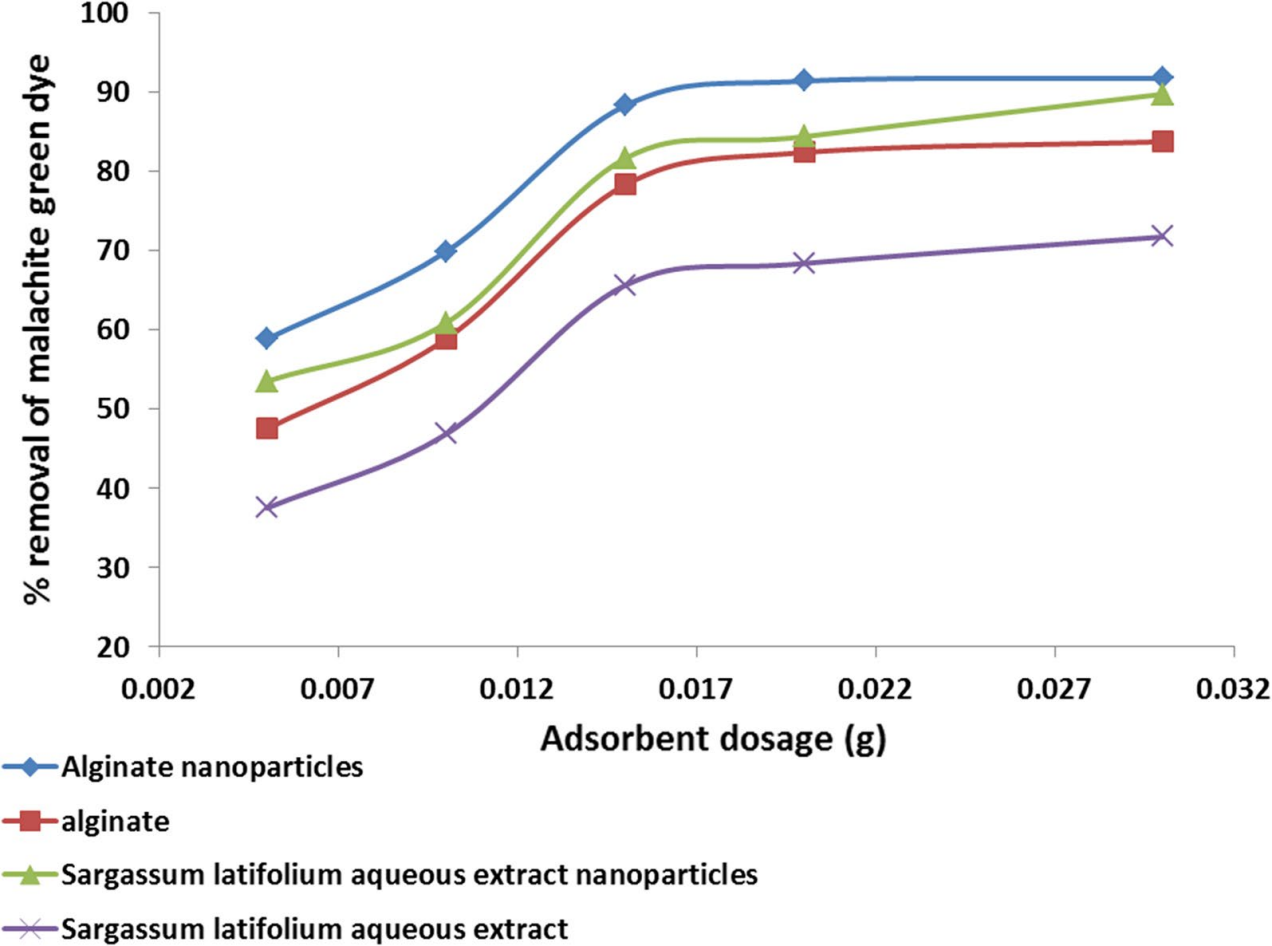
Effect of Adsorbent dose alginate, alginate silver nanoparticles, *Sargassum latifolium* aqueous extract nanoparticles and aqueous extract on bioremoval of malachite green dye

Table 3 demonstrates the effect of the adsorbent dose on the bioadsorption of malachite green dye from an aqueous solution. It can be observed from Fig. 4 that the bioadsorption percent and amount of dye adsorbed were increased as the adsorbent doses were increased from 0.005 to 0.03 g at 100 mg/L MG concentration on an equilibrium time of 3 h and pH 7. Nanoparticles showed the highest adsorption capacity. [32] reported an increase in MG removal rate by increasing adsorbents doses due to the presence of free adsorption sites and an increase in the adsorbent surface. Moreover, [33] suggested that malachite cations can be more closely bound to the negative charges on the adsorbent surface by increasing the adsorbent dosage. Increasing the amount of collisions between dye molecules and the adsorbent surface increases the removal of malachite from an aqueous solution by bioadsorption and increases the accuracy of bioadsorption. Also, [34] reported that there was a decrease in the amount of bioadsorption when the equilibrium was reached. This could be due to bioadsorption site overlapping or aggregation, leading to a reduction in the total adsorbent surface area available to MG and an increase in diffusion path length. On the other hand, [24] found that increasing the adsorbent dose, on the other hand, increases the number of free functional groups that do not attach to malachite green, reducing the bioadsorption capacity.

**Table 3:** Effect of Adsorbent dose alginate, alginate silver nanoparticles, *Sargassum latifolium* aqueous extract nanoparticles and aqueous extract on bioadsorption of malachite green dye

Adsorbent dose (g)	C_o_ (ppm)	Treatments											
		Alginate nanoparticles			Alginate			*Sargassum latifolium* aqueous extract nanoparticles			*Sargassum latifolium* aqueous extract		
		C_e_ (ppm)	Q (mg/)	Removal rate	C_e_ (ppm)	Q (mg/g)	Removal rate	C_e_ (ppm)	Q (mg/g)	Removal rate	C_e_ (ppm)	Q (mg/g)	Removal rate
0.005	100	41 ± 0.17	1176 ± 3.4	58 ± 0.97	52 ± 2	950 ± 8	48 ± 5	46 ± 1	1070 ± 8	54 ± 5	62 ± 2	750 ± 8	38 ± 5
0.01	100	30 ± 0.12	698 ± 1.2	69 ± 2.9	41 ± 5	588 ± 1.2	59 ± 2	39 ± 2	608 ± 1.2	61 ± 3	53 ± 3	468 ± 1	47 ± 5
0.015	100	11.7 ± 5	588 ± 2.8	88 ± 6	21.2 ± 1.4	521 ± 2.8	78 ± 5	18 ± 0.37	544 ± 2	82 ± 5	34 ± 1	437 ± 2	66 ± 2
0.02	100	8.6 ± 0.37	456 ± 1.8	91 ± 10	20.7 ± 2	412 ± 1.8	82 ± 10	16 ± 3	422 ± 1	84 ± 6	32 ± 2	341 ± 2	68 ± 3
0.03		8.3 ± 0.25	305 ± 10	91.723 ± 5.255	16 ± 1.2	279 ± 0.84	84 ± 6	10 ± 0.2	299 ± 0.8	89 ± 2	28 ± 0.25	239 ± 0.8	72 ± 1

C_o_: initial MG concentrations pH:7

C_e_: MG concentrations (ppm) after incubation period (150 min)

Q: biosorption capacity (mg/g)

### Effect of initial dye concentrations on bioadsorption

**Fig. 5:**
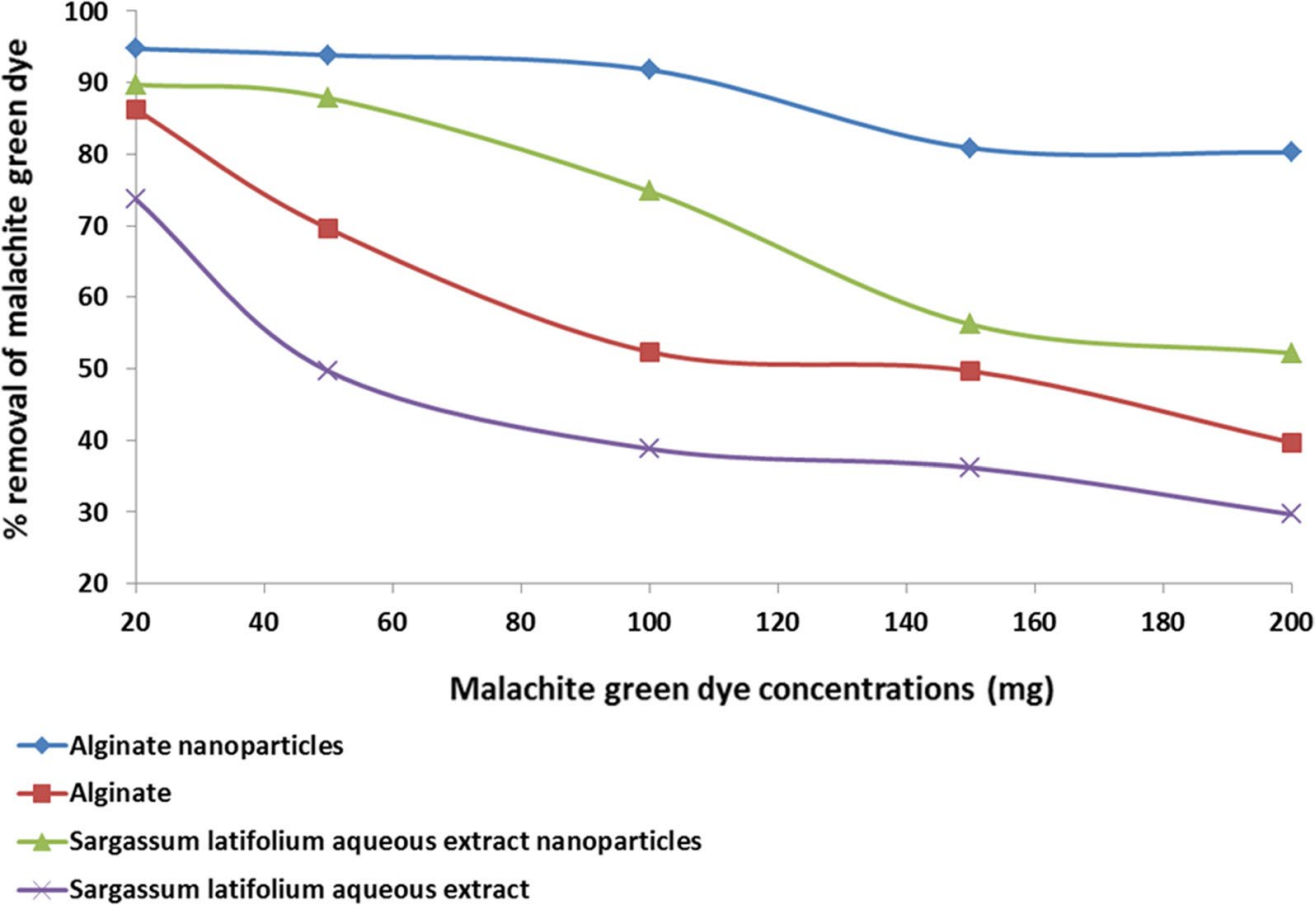
Percentage of bioadsorption of Malachite green dye by alginate nanoparticles, alginate, *Sargassum latifolium* aqueous extract nanoparticles, and *S. latifolium* aqueous extract with different malachite green dye concentrations

Adsorbate concentration is an important factor affecting the biosorption process. The present study determined the highest removal percentage at 20 mg/L and the lowest at 200 mg/L MG concentrations (Fig. 5 and Table 4). The same findings were reported by [22, 24, 35] during an adsorption study of malachite green dye using a variety of adsorbents.

**Table 4:** Effect of different malachite green concentrations on its bioadsorption by alginate nanoparticles, Alginate, *S. latifolium* aqueous extract nanoparticles and *Sargassum latifolium* aqueous extract

MG Concentrations (mg)	Treatments											
	Alginate nanoparticles (0.02 g)			Alginate (0.02 g)			*S. latifolium* aqueous extract nanoparticles (0.02 g)			*S. latifolium* aqueous extract (0.02 g)		
	C_e_ (mg)	Q (mg/g)	Removal rate	C_e_ (mg)	Q (mg/g)	Removal rate	C_e_ (mg)	Q (mg/g)	Removal rate	C_e_ (mg)	Q (mg/g)	Removal rate
20	1 ± 0.06	95 ± 1	95 ± 5	2.75 ± 0.2	86 ± 1	86 ± 2	2.05 ± 0.01	90 ± 0.05	90 ± 5	5 ± 0.01	74 ± 0.06	74 ± 0.07
50	3 ± 0.02	234 ± 4	94 ± 0.04	15 ± 4	174 ± 20	70 ± 8	6.07 ± 0.02	220 ± 12	88 ± 5	25 ± 2	124 ± 28	50 ± 3
100	8 ± 0.3	459 ± 1	92 ± 3	48 ± 17	262 ± 25	52 ± 5	25 ± 3	374 ± 10	75 ± 3	61 ± 6	194 ± 14	39 ± 6
150	29 ± 0.03	607 ± 0.15	81 ± 0.02	75 ± 11	373 ± 7	50 ± 4	66 ± 7	421 ± 16	56 ± 6	96 ± 12	272 ± 13	36 ± 8
200	40 ± 0.02	803 ± 0.12	80 ± 0.01	121 ± 19	397 ± 14	40 ± 2	96 ± 5	522 ± 13	52 ± 2	141 ± 12	297 ± 15	30 ± 6

Because there are a constant number of active sites on the adsorbent rather than an increase in the number of adsorbate molecules, the removal rate decreases as dye concentration increases [36, 37]. However [38], reported that raising the initial dye concentration may result in the formation of a repulsive interaction between adsorbate molecules, preventing them from adhering to the surface of the adsorbent.

## UV scan analysis of malachite green dye before and after treatment

**Fig. 6:**
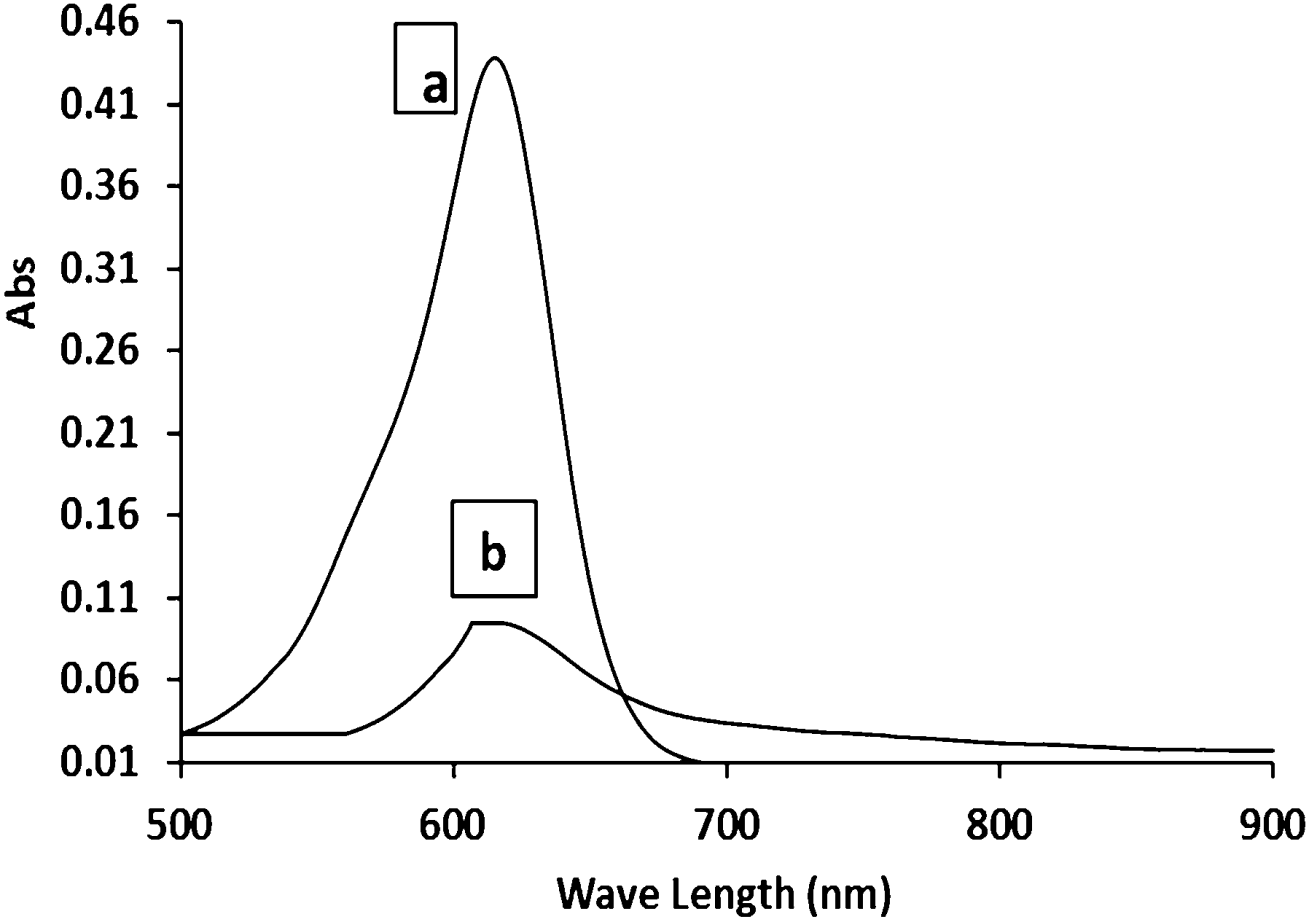
UV scan analysis of malachite green dye (100 mg/L) **a** without alginate nanoparticles **b** with alginate nanoparticles

UV—vis spectrum was performed for the initial malachite green dye and treated MG dye to show if the maximum absorption peak was changed due to biodegradation (Fig. 6**)**. The maximum absorption peak of MG dye was 615 nm. In the present study, by the addition of alginate nanoparticles (0.02 g) to MG dye (100 mg/L), we obtained decreased area under the curve in the visible range for MG dye but wasn’t a complete disappearance which could be due to bioadsorption. [35] reported that after bioadsorption of dye the UV signals tend to stay constant with a lower area than those of the initial dye.

## Fourier transform infrared spectrometry (FT-IR)

**Fig. 7:**
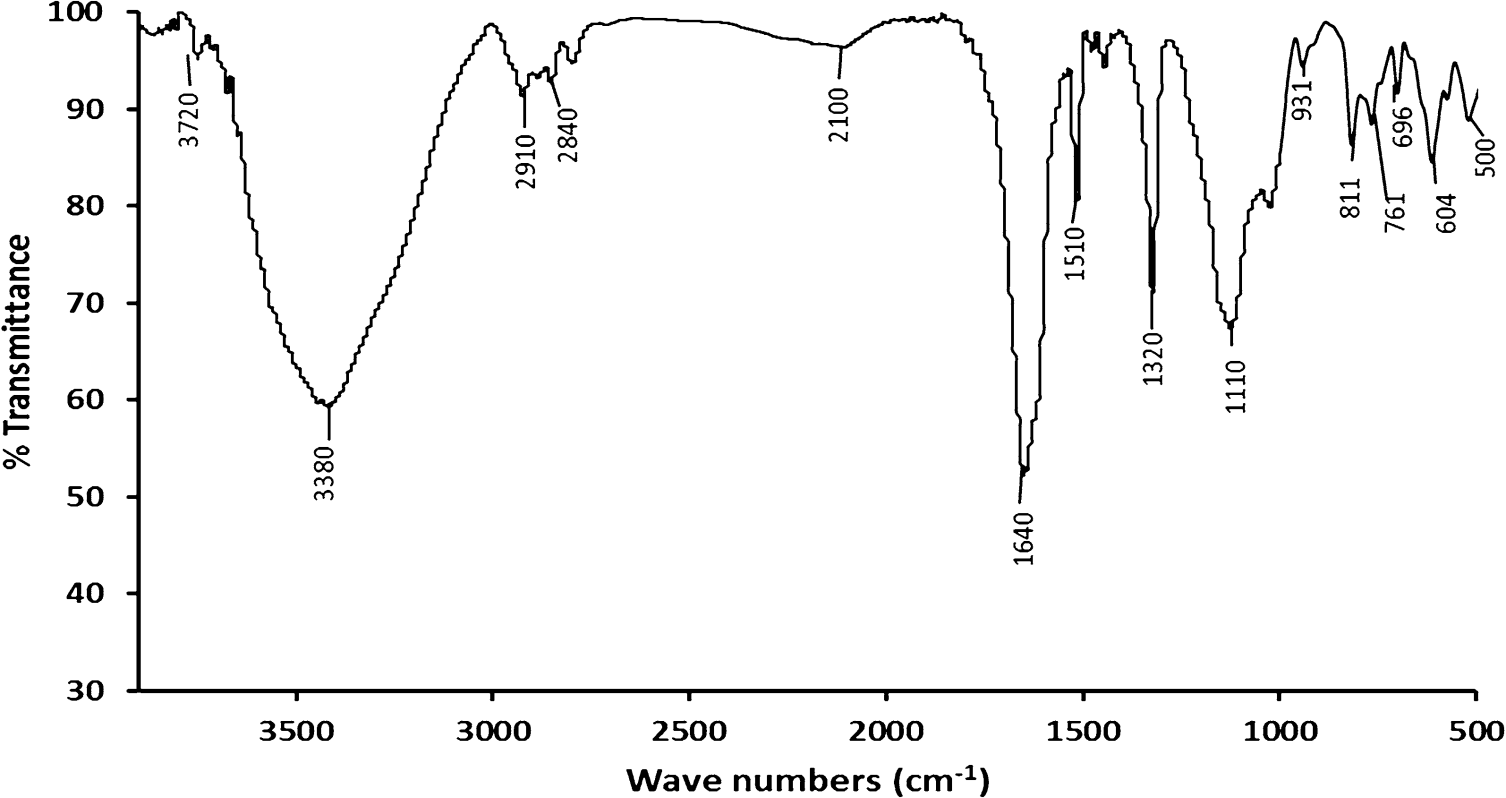
Fourier Transform Infrared Spectrometry (FT-IR) of alginate silver nanoparticles after bioadsorption of malachite green dye

Figure 7 presents the spectra obtained for alginate silver nanoparticles after the bioadsorption of MG. While alginate silver nanoparticles before adsorption were obtained in our previous research [14]. Table 5 presents the shifts suffered by the bands after the bioadsorption of dye by alginate silver nanoparticles. The bands located at 3850, 3721, 3415, 2911, and 2480 cm^−1^ assigned to hydroxyl groups of adsorbents, their transmittance decreased considerably, suggesting that these groups are probably involved in the binding of malachite green to the adsorbent. Also, [21] reported that this shift in peak values is due to the formation of the chemical bond between functional groups present in adsorbent and malachite green dye. On the other hand, [39] reported that when malachite green was dissolved in an aqueous solution, they were positively charged and showed attraction to the OH group from adsorbents. Similar observations were obtained by [20, 21, 24] on the adsorption of malachite green dye by many adsorbents.

**Table 5:** Shifting observed in the bands after bioadsorption of malachite green dye by alginate nanoparticles

ALG—AgNPs IR bands (cm^−1^)	ALG—AgNPs with malachite green dye IR bands (cm^−1^)
3859	Disappear
3721	3720
3415	3380
2911	2910
2841	2840
2480	2100
1619	1640
1411	Disappear
1115	1110
898	811
696	696
612	604
	1510
	931
	761

## Adsorption isotherm

**Fig. 8:**
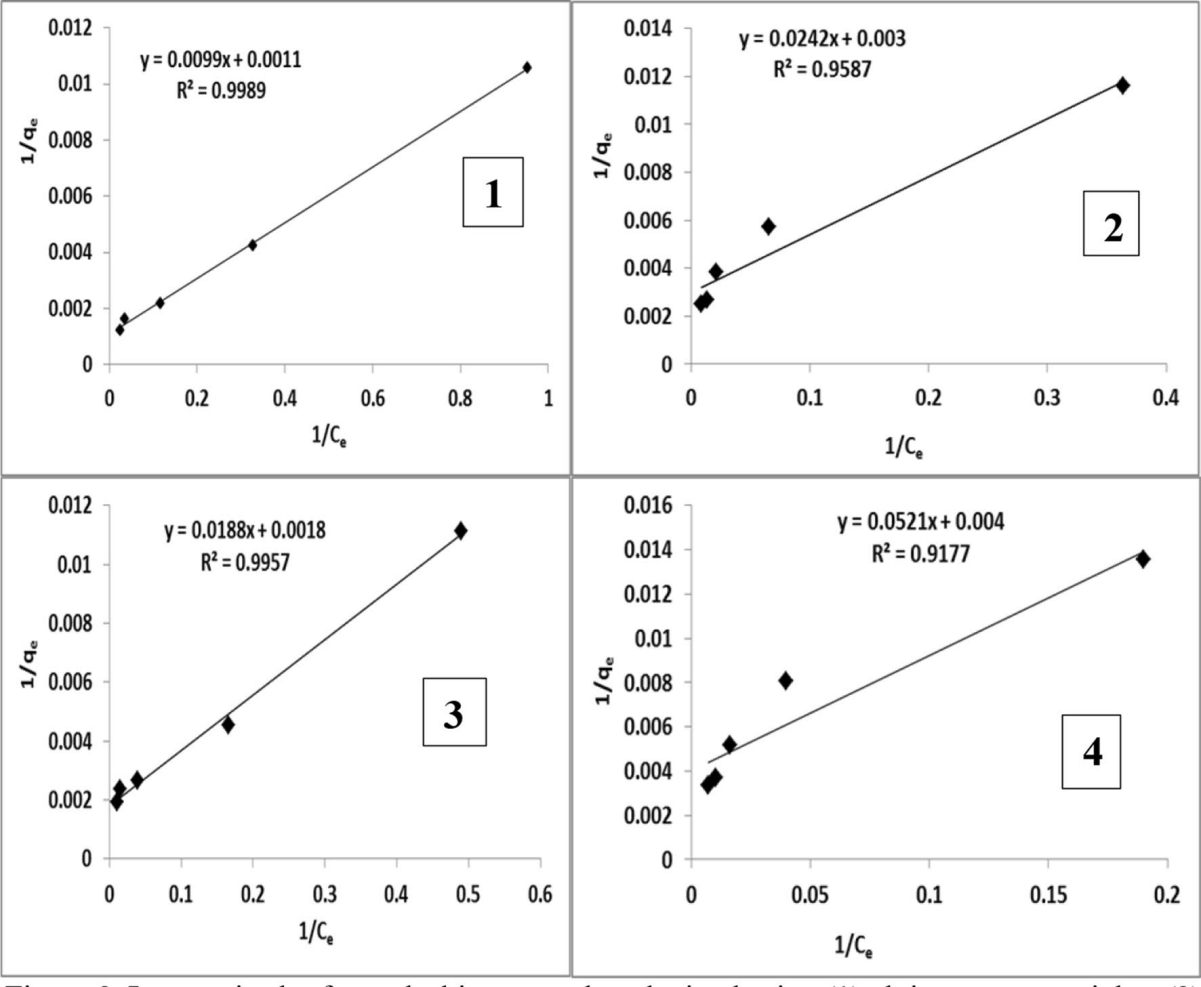
Langmuir plot for malachite green dye obtained using (1) alginate nanoparticles, (2) alginate, (3) *Sargassum latifolium* aqueous extract nanoparticles, and (4) *S. latifolium* aqueous extract

**Fig. 9:**
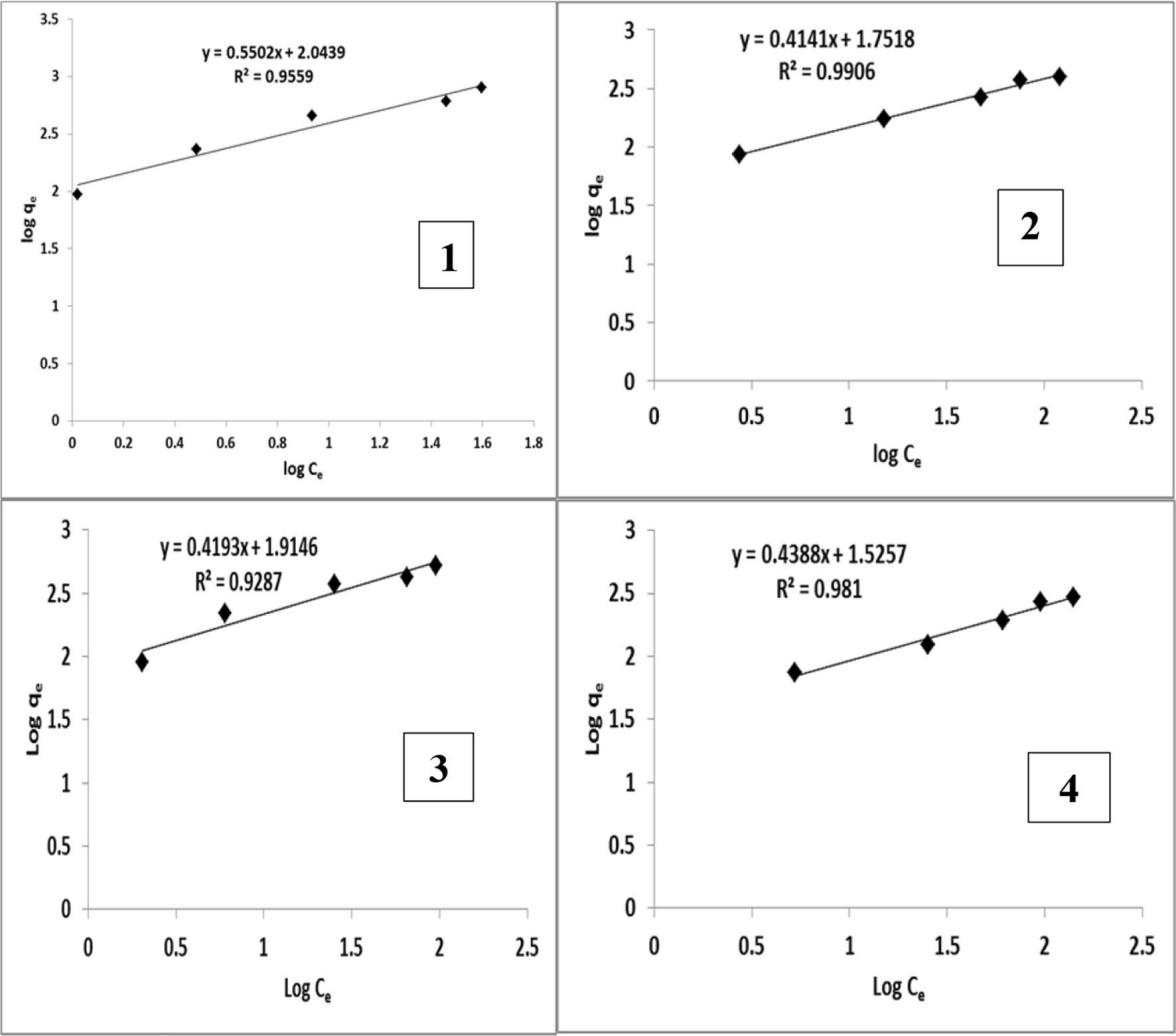
Freundlich plot for malachite green dye obtained using (1) alginate nanoparticles, (2) Alginate, (3) *Sargassum latifolium* aqueous extract nanoparticles and (4) *S. latifolium* aqueous extract

The collected equilibrium data for the studied malachite green dye over the concentration range from 20 to 200 mg/L at 25 °C were fitted to Langmuir isotherm (Fig. 8) for alginate nanoparticles and *S. latifolium* aqueous extract nanoparticles and also fitted to Freundlich isotherm (Fig. 9) for alginate and *S. latifolium* aqueous extract. Table 6 shows Langmuir and Freundlich’s parameters obtained using alginate nanoparticles, Alginate, *S. latifolium* aqueous extract nanoparticles, and *S. latifolium* aqueous extract. It can be observed that the degree of dye removal by alginate and *Sargassum latifolium* aqueous extract increased after the formation of nanoparticles. Sodium alginate nanoparticles showed the highest removal rate followed by aqueous extract nanoparticles.

**Table 6:** Langmuir and Freundlich parameters obtained using alginate nanoparticles, Alginate, *Sargassum latifolium* aqueous extract nanoparticles and *S. latifolium* aqueous extract

Treatments	Parameters							
	Langmuir isotherm constants				Freundlish isotherm constants			
	Q_max_ (mg/g)	K_L_ (L/mg)	R_L_	R^2^	1/n	n	K_f_ (mg/g)	R^2^
Alginate nanoparticles	909	0.11	0.31–0.04	0.998	0.55	0.81	110	0.955
Alginate	333	0.12	0.2874–0.038	0.958	0.41	0.41	56	0.990
*S. latifolium* Aqueous extract nanoparticles	556	0.09	0.3430–0.049	0.995	0.41	0.38	82	0.928
*S. latifolium* Aqueous extract	250	0.05	0.4565–0.077	0.917	0.8	0.24	19	0.981

Q_max_: the maximum monolayer coverage capacity

K_L_: Langmuir constant isotherm

R_L_: separation factor or equilibrium parameter

K_f_: Freundlich isotherm constant

n: adsorption intensity or surface heterogeneity

Langmuir and Freundlich’s adsorption isotherm models were used to analyze the experimental results. According to correlation coefficient values of alginate, alginate nanoparticles, aqueous extract, and aqueous extract nanoparticles in Langmuir (R^2^ = 0.958, 0.998, 0.917, and 0.995, respectively) and Freundlich models (R^2^ = 0.990, 0.95, 0.981 and 0.92 respectively) the obtained data were fitted to Langmuir and Freundlich isotherm models which have been applied to adsorbents with heterogeneous surfaces and consider multilayer sorption. According to [40], the adsorption process in the Langmuir model proceeded uniformly and on a homogenous surface as an bioadsorption monolayer with no interaction between adsorbed molecules. In the Freundlich model, multilayer adsorption is considered to occur with a non-uniform energy distribution of the adsorption sites as well as interference from adsorbed ions. Also, [41] also stated that this model's constants are K_f_ and n, which reflect the absorption capacity and intensity, respectively.

Consequently, Our Values of 1/n between 0 and 1 indicate the heterogeneity of the adsorbent. Obtained 1/n for alginate, alginate nanoparticles, aqueous extract, and aqueous extract nanoparticles were 0.414, 0.550, 0.805, and 0.419, respectively. Moreover, [32] reported that an adsorption isotherm represents the equilibrium relationship between the adsorbate concentration in the liquid phase and that on the adsorbent’s surface at a given condition. It also illustrates how adsorbed materials interact with the adsorbent, giving a comprehensive view of the bioadsorption process, and indicating that the removal rate and heterogeneity of the adsorbent are favorable.

## Conclusion

Sodium alginate, *S. latifolium* aqueous extract, and their silver nanoparticles are economical and effective bioadsorbent for the bioremoval of malachite green dye from aqueous solutions. Moreover, adsorption is influenced by various parameters such as initial pH, contact time, adsorbent quantity, and initial dye concentrations. The maximum removal rate was obtained (69.8%) by using alginate silver nanoparticles at pH 7 with an adsorbent dose of 10 mg and with an agitation time of 240 min. UV scan analysis and FTIR confirms the adsorption process. Data obtained were fitted to Langmuir and Freundlich isotherms.

## Data Availability

The datasets generated and analyzed during the current study are available from Nagwa I. Hassan.
